# Understanding the Association of Plasmid Incompatibility Groups With Variable Antimicrobial Resistance Genotypes in Bacteria

**DOI:** 10.1002/mbo3.70187

**Published:** 2025-12-09

**Authors:** Hannay Crystynah Almeida de Souza, Pedro Panzenhagen, Anamaria Mota Pereira dos Santos, Ana Beatriz Portes, Arlen Carvalho de Oliveira Almeida, Carlos Adam Conte Junior

**Affiliations:** ^1^ Center for Food Analysis (NAL), Technological Development Support Laboratory (LADETEC) Federal University of Rio de Janeiro (UFRJ), Cidade Universitária Rio de Janeiro Brazil; ^2^ Laboratory of Advanced Analysis in Biochemistry and Molecular Biology (LAABBM), Department of Biochemistry Federal University of Rio de Janeiro (UFRJ), Cidade Universitária Rio de Janeiro Brazil; ^3^ Graduate Program in Biochemistry (PPGBq), Institute of Chemistry (IQ) Federal University of Rio de Janeiro (UFRJ), Cidade Universitária, Rio de Janeiro Rio de Janeiro Brazil; ^4^ Graduate Program in Veterinary Hygiene (PGHIGVET), Faculty of Veterinary Medicine Fluminense Federal University (UFF), Vital Brazil Filho Niterói Rio de Janeiro Brazil; ^5^ Laboratory of Microorganism Structure, Department of General Microbiology, Institute of Microbiology Paulo de Góes (IMPG) Federal University of Rio de Janeiro 21941‐902 Rio de Janeiro Brazil; ^6^ Graduate Program in Food Science (PPGCAL), Institute of Chemistry (IQ) Federal University of Rio de Janeiro (UFRJ), Cidade Universitária, Rio de Janeiro Rio de Janeiro Brazil; ^7^ Analytical and Molecular Laboratory Center (CLAn), Institute of Chemistry (IQ) Federal University of Rio de Janeiro (UFRJ), Cidade Universitária Rio de Janeiro Brazil

**Keywords:** antimicrobial resistance dissemination, genetic mobility, multi‐replicon plasmids, plasmid incompatibility groups

## Abstract

Plasmids play an essential role in the spread of antimicrobial resistance (AMR) by facilitating the horizontal transfer of resistance genes between bacterial environments. However, large‐scale investigations into the association between plasmid incompatibility groups (Inc groups) and specific resistance profiles remain limited. In this study, we analyzed 28,047 plasmid sequences from publicly available whole‐genome sequencing data sets, identifying incompatibility groups in 11,288 plasmids using in silico replicon typing. Our results revealed that the majority of plasmids harbored a single replicon, while a substantial fraction carried multiple replicons, predominantly two. We evaluated the relationship between plasmid replicon spillovers and their role in the spread of resistance genes. Our results revealed that plasmids with five replicons have a significantly higher resistance potential (60%) compared to plasmids with fewer replicons, decreasing their adaptability and propensity for cointegration, which facilitates horizontal gene transfer. Among the resistance‐associated plasmids, the IncF, IncI, and IncH families were predominant and acted as effective carriers of resistance genes. Comparative analyses between resistant and non‐resistant plasmids did not reveal a clear visual pattern of association between the most prevalent Inc groups and specific antimicrobial classes, indicating that such relationships are shaped by contextual factors, including selective instructions, bacterial host diversity, and distribution. These findings highlight the complexity of the spread of plasmid‐mediated AMR and highlight the need for integrated genomic and epidemiological approaches to better understand the ecological and evolutionary dynamics that influence the spread of resistance genes.

## Graphical abstract

Multi‐replicon plasmids, particularly those belonging to the IncF, IncI, and IncH families, act as major vectors of antimicrobial resistance genes. Although no clear association was found between incompatibility groups and specific antibiotic classes, plasmids with more replicons exhibited higher resistance potential, highlighting their role in AMR dissemination.

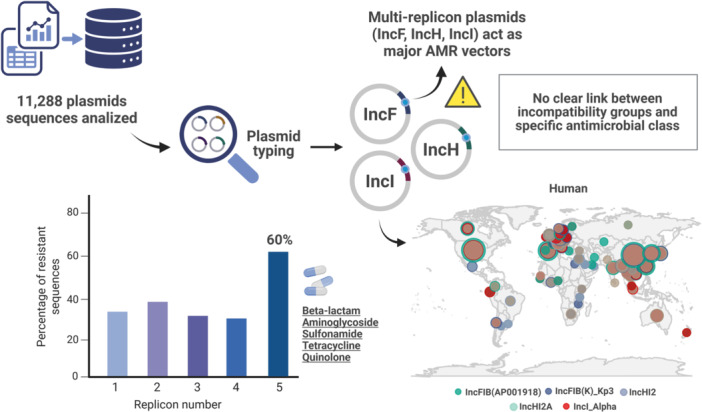

## 1 Introduction

Antimicrobial‐resistant bacteria represent a serious public health hazard in the 21st century, threatening the effectiveness of conventional treatments and leaving many infections without viable therapeutic options (Salam et al. 2023). Although traditional antibiotics are essential to modern medicine, their efficacy has been increasingly compromised by the remarkable ability of bacteria to rapidly evolve and disseminate resistance genes, particularly through mobile genetic elements (MGEs) such as plasmids (Davies and Oxlade 2021). These plasmids can spread swiftly via horizontal gene transfer across bacterial populations, including in high‐risk settings such as hospitals (Partridge et al. 2018). Notably, plasmids often carry multiple resistance genes, contributing to the emergence of multidrug‐resistant strains and exacerbating the global antimicrobial resistance (AMR) crisis (Lerminiaux and Cameron 2019). Without the implementation of effective control strategies, this scenario may lead to an estimated 10 million deaths per year by 2050, in addition to a projected annual loss of up to US$3.4 trillion in global GDP (O'neill 2016).

Plasmids can be classified into different incompatibility groups based on gene variations in the plasmid structure's replication and regulation modules (Novick 1987). Some incompatibility groups may include several subgroups that are incompatible with each other. The subgroups of the IncF, IncL/M, IncA/C, IncI, and IncH groups, for example, are the most common plasmids in *Enterobacterales* (Carattoli 2009). Several studies have shown that many of these plasmids can carry multiple AMR genes (Liang et al. 2022; Mitra et al. 2021; Sethuvel et al. 2019; Han et al. 2012). For instance, IncF family plasmids are closely associated with the carriage of AMR genes, including those encoding extended‐spectrum β‐lactamases (ESBL) such as *bla*_CTX‐M‐14_ and *bla*_TEM‐191_, aminoglycoside (*aph(3”)‐lb*, *aph(6)‐ld*), quinolone (*qnrS1*), macrolide (*mph*(A)), sulfonamide (*sul1*), and trimethoprim (*dfrA27*) (Du et al. 2022; Douarre et al. 2020; Orlek et al. 2023; Wang et al. 2021). Furthermore, IncF plasmid subgroups are notable for their ability to promote co‐integration, a process in which different plasmids merge into a single replicon (Douarre et al. 2020). This ability increases plasmid structural complexity and enhances the efficiency of disseminating multiple resistance genes, which poses a significant challenge for AMR control (Orlek et al. 2023).

Multi‐replicon plasmids have attracted great interest in the scientific community due to their complex structure and the ability to integrate multiple replicons into a single plasmid backbone (Douarre et al. 2020; Orlek et al. 2023; Wang et al. 2021). These plasmids often carry multiple AMR genes, making them interesting targets for AMR mitigation (Orlek et al. 2023; Wang et al. 2021; Chen et al. 2024). In China, for example, a multi‐replicon plasmid (pHS091147) was identified in a multidrug‐resistant *Klebsiella pneumoniae* isolate (Tang et al. 2017). This plasmid had a mosaic genetic structure with a diversity of resistance genes to last‐resort antimicrobials, such as carbapenems (*bla*_KPC‐2_), as well as genes associated with resistance to sulfonamide (*sul1*) and trimethoprim (*dfrA25*) and ESBL (*bla*_CTX‐M‐14_ and *bla*_TEM‐1_) (Tang et al. 2017). The ability of a single plasmid to carry all these resistance genetic elements raises concerns about the spread of this complex plasmid structure.

Regarding their spreading characteristics, the mobilization of plasmids among the bacterial population is mainly governed by the incompatibility groups to which they belong, regulating their ability to coexist in the same bacterial cell (Couturier et al. 1988). Plasmids from the same incompatibility group share similar replication and partitioning modules, which prevents them from coexisting in the same bacterial cell due to competition between the replication machinery (Novick 1987). This restriction, which regulates the diversity of plasmids within a single cell, still provides selective pressure for more adapted plasmids that confer evolutionary advantages to certain bacteria, such as those carrying AMR genes (Hoang et al. 2022).

Our understanding of the dynamics of AMR dissemination via plasmids within bacterial populations is continually evolving, necessitating broader association studies that correlate AMR with plasmid incompatibility groups. A particular study compiling literature data on plasmids and their relationships with AMR demonstrated that certain incompatibility groups are strongly linked to the carriage of specific resistance genes (Rozwandowicz et al. 2018). For instance, IncA/C plasmids were linked to multidrug resistance, carrying genes such as *bla*_TEM_, *bla*_CMY_, *bla*_OXA_, *sul1*, *sul2*, *aadB*, *strA*, and *tet(A)*. IncF plasmids were associated with ESBL resistance genes and carbapenemase‐encoding genes. IncP plasmids were connected to genes conferring resistance to ESBLs, sulfonamides, aminoglycosides, and tetracyclines (Rozwandowicz et al. 2018). To date, studies have often been limited by small sample sizes and localized data and have been based on conventional molecular techniques, with low capture of the complexity of AMR dissemination across diverse bacterial populations (Lindsey et al. 2009; Villa et al. 2010; Kim et al. 2011; Valverde et al. 2009).

Despite significant progress in understanding plasmid‐mediated AMR, many gaps remain in linking specific plasmid incompatibility groups with resistance patterns on a broader scale. With the availability of publicly accessible large‐scale genomic databases and advanced bioinformatics tools, there is a vast opportunity to overcome these limitations and discover novel associations. In this study, we investigate the existence of correlations between plasmid incompatibility groups (Inc) and AMR profiles using an extensive data set of plasmid sequences from diverse bacterial populations across multiple geographic regions. We categorized plasmids into incompatibility groups and assessed their correlation with specific resistance genes. Our analysis focused on identifying patterns of resistance dissemination facilitated by single‐ and multi‐replicon plasmids, with particular emphasis on understanding the adaptive potential of plasmids harboring multiple replicons. Through statistical and computational approaches, we evaluated the prevalence of resistance genes within different Inc groups and examined whether these groups exhibited visually clear associations with specific antimicrobial classes. This comprehensive exploration provides novel insights into the role of plasmids as vectors of multidrug resistance, offering a foundation for the development of alternative therapeutic strategies, such as targeting plasmid‐mediated resistance pathways, and for designing effective policy interventions to mitigate the spread of AMR in both clinical and environmental settings.

## 2 Materials and Methods

### 2.1 Data Collection and Screening

On April 25, 2024, we collected 59,895 plasmid sequences from the PLSDB (Plasmid Database) v. 2023_11_03_v2, available at https://ccb‐microbe.cs.uni‐saarland.de/plsdb. The screening procedure was performed by selecting only complete plasmid sequences. Inclusion criteria based on the available metadata required information regarding (i) location, (ii) host, and (iii) taxonomic units (class, order, family, and genus).

### 2.2 *In Silico* Detection and Plasmid Typing

We used ABRicate software to initiate a large‐scale search for AMR genes and replicons from all eligible plasmid genomes. The ResFinder v 4.5.0 (Zankari et al. 2012) and PlasmidFinder v 2.0.1 (Carattoli et al. 2014) databases were used due to their broad coverage in identifying resistance genes and plasmid replicons, respectively. The parameters used for ABRicate analysis were as follows: minimum identity (minid) of 95% and minimum coverage (mincov) of 60%. These default settings provide a highly specific and reliable detection of resistance genes, as demonstrated in a previous study that reported a 99.74% concordance with phenotypic antimicrobial susceptibility tests (Zankari 2014). These parameters ensure a balance between sensitivity and specificity, minimizing false positives while maintaining high accuracy.

### 2.3 Distribution of Resistance Genes

The distribution of resistance genes was analyzed within the incompatibility groups as mentioned above. Data were normalized to avoid bias due to variations in the number of sequences between incompatibility groups. Evaluation of resistance distribution was performed by calculating the relative frequency of resistance genes associated with each antibiotic class within each incompatibility group, allowing comparison of proportions rather than absolute numbers. The calculation was the number of plasmid sequences resistant to a given class of antibiotics divided by the total number of plasmid sequences within the respective incompatibility group.

### 2.4 Statistical Analysis

#### 2.4.1 Analysis of Host Range by Plasmid Incompatibility

For each plasmid incompatibility group (Inc), host range was assessed, defined as the diversity of bacterial genera carrying plasmids of that group. First, host richness was calculated, defined as the number of unique genera associated with each Inc group. Next, the Shannon diversity index was calculated for each group, considering the distribution of genus abundances within each Inc. To control for potential biases due to differences in plasmid counts per group, normalized versions of richness and Shannon metrics were also calculated by dividing each value by the total number of plasmids in the respective Inc group. All analyses were performed in RStudio (version 4.4.0) using the packages readxl for data import, dplyr for data manipulation, and vegan for calculating the Shannon index.

#### 2.4.2 Principal Component Analysis (PCA)

Statistical analysis was conducted using plasmid sequences from the most frequent incompatibility groups associated with AMR. The primary objective was to explore potential interrelationships among the analyzed variables, including resistance patterns and plasmid‐associated characteristics such as host, geographic location, genus, and species. PCA was performed to identify the most relevant variables and assess the magnitude of their contribution to data variance.

To minimize bias in the model, non‐informative variables, such as unique identifiers (e.g., accession numbers), were excluded before the analysis. Categorical variables, including incompatibility group, genus, species, location, and host, were converted into factors and processed via binary encoding (one‐hot encoding) using the ggplot package in R. This procedure ensured the consistent integration of categorical and numerical variables.

PCA was conducted using the FactoMineR package in R, with the data previously encoded and standardized to ensure comparability between variables. The first two principal components (PC1 and PC2), which accounted for the largest proportion of total variance, were selected for further interpretation and visualization. To facilitate interpretation and highlight the most relevant variables, a selection was made based on the contribution values (contrib) calculated by PCA, retaining the 20 variables with the highest influence on the principal components.

#### 2.4.3 Statistical Analysis of Associations Between Plasmid Incompatibility Groups and AMR Classes

To investigate possible associations between plasmid incompatibility groups (Inc) and AMR classes, the proportion of resistant plasmids was calculated for each combination of Inc group and antibiotic class. Based on these data, statistical association tests were applied to assess whether the presence of resistance was significantly related to the plasmid type. For combinations with adequate expected counts, the chi‐square test was used, while for combinations with expected values less than five, Fisher's exact test, simulated with 10,000 replicates, was applied, ensuring greater precision in the results.

Each Inc group was compared individually against all other plasmids for each antibiotic class, allowing for the identification of specific associations. The p‐values obtained are presented in Supporting Information S1: Table S4, enabling a detailed evaluation of the exploratory associations between plasmids and AMR.

## 3 Results

### 3.1 Data Collection and Screening

From the 59,895 plasmid sequences available in the PLSDB database, 56,161 were selected based on the completeness of their sequences. Of these, 28,047 plasmids met the inclusion criteria, as defined by the available metadata. Among these, incompatibility groups were identified in 11,288 plasmids. This final data set was subsequently used for further analyses, including screening for AMR genes.

### 3.2 *In Silico* Analyses: Characterization of Plasmids Regarding Incompatibility Groups

#### 3.2.1 General Incompatibility Group Distribution

Across the analyzed plasmids, a total of 432 distinct incompatibility groups were identified. This wide diversity led us to focus on the most frequent incompatibility groups to ensure robust statistical analyses and meaningful interpretations. To achieve this, we established a minimum threshold of 4% frequency within the database. This threshold allowed the inclusion of the following groups, along with the respective number of genomes assigned: IncFIB (AP001918) (1275 genomes, 11%), IncFIB(K)Kpn3 (764 genomes, 7%), IncR (624 genomes, 6%), IncHI2A (500 genomes, 5%), IncFIA (610 genomes, 5%), IncA/C2 (405 genomes, 4%), IncFII (469 genomes, 4%), IncHI2 (498 genomes, 4%), and IncI1Alpha (484 genomes, 4%). This selection ensures the analysis of incompatibility groups with substantial representation, thereby reducing the potential for bias or overinterpretation of less frequent groups. The distribution analysis of incompatibility groups revealed that the IncF group was the most prevalent. This group, which includes the subgroups IncFIA, IncFIB (AP001918), IncFIB (K)Kpn3, and IncFII, accounted for 55.4% of the identified variants. In contrast, the IncHI, IncR, IncI, and IncA/C groups were present at lower frequencies, accounting for 17.7%, 11.1%, 8.6%, and 7.2%, respectively.

#### 3.2.2 Incompatibility Group Distribution According to the Number of Replicons

Of the genomes analyzed, 8044 (71.28%) presented a single replicon, while 3244 (28.74%) contained multiple replicons. Among the genomes with multiple replicons, the majority harbored two replicons (2,394 genomes, representing 73.84%), followed by three replicons (654 genomes, 20.15%), four replicons (181 genomes, 5.58%), and five replicons (15 genomes, 0.46%). To investigate the relationship between incompatibility groups and AMR, we analyzed the frequency distribution of incompatibility groups among non‐resistant (*n* = 7275) (Table 1) and resistant plasmids (*n* = 4013) (Table 2) categorized by the number of replicons.

**Table 1:** Incompatibility group distribution based on number of replicons in non‐resistant plasmids.

Non‐resistant plasmids	Inc group	Genomes	%
Single replicon (5205)			
IncFIB (K)_Kpn3	IncF	356	6,84
IncI1_Alpha	IncI	275	5,28
IncA/C2	IncA	241	4,63
IncFII	IncF	232	4,46
IncFIB(AP001918)	IncF	225	4,32
IncR	IncR	203	3,90
IncX3	IncX	190	3,65
Two replicons (1505)			
IncFIB(AP001918)	IncF	317	21,06
IncHI2A	IncH	256	17,01
IncHI2	IncH	255	16,94
IncR	IncR	169	11,23
IncFIA	IncF	165	10,96
IncFIB(K)Kpn3	IncF	122	8,11
IncFII(pHN7A8)_pHN7A8	IncF	103	6,84
IncFII(S)	IncF	88	5,85
IncFIB(S)	IncF	80	5,32
IncHI1B_pNDM‐MAR	IncH	80	5,32
IncFIB(Mar)_pNDM‐Mar	IncF	74	4,92
IncFIC(FII)	IncF	69	4,58
IncFIA(HI1)_HI1	IncF	69	4,58
rep19_3_CDS20(pSJH901)	Rep	68	4,52
rep5a_repSAP001(pN315)	Rep	59	3,92
IncFII	IncF	53	3,52
Three replicons (436)			
IncFIB (AP001918)	IncF	199	45,64
IncFIA	IncF	139	31,88
Col156	Col	71	16,28
IncN	IncN	61	13,99
IncQ1	IncQ	60	13,76
IncHI2A	IncH	58	13,30
IncHI2	IncH	58	13,30
IncFIA(HI1)_HI1	IncF	50	11,47
IncFIA(HI1)_HI1	IncF	50	11,47
IncFII(pRSB107)	IncF	46	10,55
IncFII(29)_pUTI89	IncF	45	10,32
IncFIC (FII)	IncF	42	9,63
IncHI1A	IncH	35	8,03
IncHI1B (R27)_R27	IncH	35	8,03
rep16_2_CDS6 (pSJH101)	Rep	32	7,34
IncFII	IncF	22	5,05
IncX1	IncX	22	5,05
rep5a_repSAP001(pN315)	Rep	19	4,36
IncFIB (K)_Kpn3	IncF	18	4,13
IncFII(pHN7A8)_pHN7A8	IncF	18	4,13
IncR	IncR	18	4,13
Four replicons (123)			
IncFIB (AP001918)	IncF	75	60,98
IncFIA	IncF	70	56,91
Col156	Col	55	44,72
IncFII (pRSB107) _pRSB107	IncF	48	39,02
IncQ1	IncQ	45	36,59
IncFIA (HI1)_HI1	IncF	29	23,58
IncHI1A	IncH	24	19,51
IncHI1B (R27)_R27	IncH	24	19,51
IncN	IncN	14	11,38
IncFII(pAMA1167‐NDM‐5)	IncF	11	8,94
IncFII	IncF	9	7,32
IncFIB(K)_Kpn3	IncF	8	6,50
IncHI2A	IncH	8	6,50
IncHI2	IncH	8	6,50
IncR	IncR	8	6,50
IncX1	IncX	7	5,69
Five replicons (6)			
rep20_12_repA (SAP105B)	Rep	2	33,33
rep22_1a_repB(pUB110)	Rep	2	33,33
rep16_2_CDS6(pSJH101)	Rep	1	16,67
rep18b_2_repA(pEF418)	Rep	1	16,67
rep19_3_CDS20(pSJH901)	Rep	1	16,67
rep21_rep(pWBG754)	Rep	1	16,67
rep22_1b_repB(pAMalpha1)	Rep	1	16,67
rep2_orf1(pRE25)	Rep	1	16,67
rep5a_repSAP001(pN315)	Rep	1	16,67
rep6_repA(pS86)	Rep	1	16,67
rep7a_16_repC (Cassette)	Rep	1	16,67
rep7a_8_ORF11(pRE25)	Rep	1	16,67
repUS22_1_rep (SAP015B)	Rep	1	16,67

**Table 2:** Incompatibility group distribution based on number of replicons in resistant plasmids.

Resistant plasmids	Inc group	Genomes	%
Single replicon (2839)			
IncFIB (K) Kpn3	IncF	195	6,87
IncI1_Alpha	IncI	181	6,38
IncFIB (AP001918)	IncF	122	4,30
IncA/C2	IncA/C	119	4,19
IncFII	IncF	113	3,98
IncR	IncR	100	3,52
Two replicons (889)			
IncFIA (AP001918)	IncF	205	23,06
IncHI2A	IncH	149	16,76
IncHI2	IncH	148	16,65
IncR	IncR	111	12,49
IncFIA	IncF	100	11,25
IncFII (pN7A8)_pN7A8	IncF	81	9,11
IncFIC (FII)	IncF	63	7,09
IncFIB(K)_Kpn3	IncF	56	6,30
rep16_2_CDS6 (pSJH101)	Rep	49	5,51
IncFII(S)	IncF	47	5,29
IncFIA (HI1) _HI1	IncF	44	4,95
IncFIB(S)	IncF	44	4,95
rep5a_repSAP001(pN315)	Rep	32	3,60
Three replicons (218)			
IncFIB (AP001918)	IncF	92	42,20
IncFIA	IncF	67	30,73
Col156	Col	38	17,43
IncN	IncN	29	13,30
IncFIA (HI1)_HI1	IncF	27	12,39
IncHI2A	IncH	21	9,63
IncFII(29)_pUTI89	IncF	20	9,17
IncFII(pRSB107)_pRSB107	IncF	19	8,72
IncHI1A	IncH	19	8,72
IncHI1B(R27)_R27	IncH	19	8,72
IncQ1	IncQ	18	8,26
rep16_2_CDS6	Rep	18	8,26
IncFIC (FII)	IncF	15	6,88
IncR	IncR	14	6,42
IncX1	IncX	13	5,96
rep2_orf1(pRE25)	Rep	13	5,96
repUS43 – CDS12738 (DOp1)	Rep	10	4,59
rep7a_8_ORF11(pRE25)	Rep	9	4,13
IncFIB(K)_Rpn3	IncF	9	4,13
IncFII (pHN7A8)_pHN7A8	IncF	9	4,13
IncFII	IncF	9	4,13
rep22_1b_repB (pAMalpha1)	Rep	9	4,13
rep7a_17_CDS4 (pS194)	Rep	8	3,67
rep5a_1_repSAP001(pN315)	Rep	8	3,67
Four replicons (58)			
IncFIB (AP001918)	IncF	36	62,07
IncFIA	IncF	34	58,62
IncQ1	IncQ	26	44,83
IncFII(pRSB107)	IncF	24	41,38
Col156	Col	23	39,66
IncFIA (HI1)_HI1	IncF	14	24,14
IncHI1A	IncH	13	22,41
IncHI1B (R27)	IncH	13	22,41
IncFII (pAMA1167‐NDM‐5)	IncF	10	17,24
IncHI2A	IncH	4	6,90
IncHI2	IncH	4	6,90
IncN	IncN	3	5,17
IncX1	IncX	3	5,17
rep20_12_repA(SAP105B)	Rep	3	5,17
rep7a_16_repC (cassette)	Rep	3	5,17
Five replicons (9)			
rep20_12_repA (SAP105B)	Rep	4	44,44
rep22_12_repB (pUB110)	Rep	4	44,44
rep5a_1_repSAP001 (pN315)	Rep	3	33,33
rep7a_16_repC (cassette)	Rep	3	33,33
repUS22_1_rep (SAP015B)	Rep	3	33,33
IncX1	IncX	3	33,33
IncN	IncN	2	22,22
IncHI1B (R27)_R27	IncH	2	22,22
IncHI1A	IncH	2	22,22
IncFIA	IncF	2	22,22
IncFIA (HI1)_HI1	IncF	2	22,22
IncFIC (FII)	IncF	1	11,11
IncFII(S)	IncF	1	11,11
IncFII (pHN7A8)_pHN7A8	IncF	1	11,11
IncFII (pRSB107)_pRSB107	IncF	1	11,11
IncHI2A	IncH	1	11,11
IncHI2	IncH	1	11,11
Col156	Col	1	11,11
IncA/C2	IncA/C	1	11,11
IncQ1	IncQ	1	11,11
rep16_2_CDS6 (pSJH101)	Rep	1	11,11
rep19_3_CDS20 (pSJH901)	Rep	1	11,11
rep21_1_rep (pWBG754)	Rep	1	11,11

Among Non‐Resistant Plasmids with a single replicon (*n* = 5,205), the most prevalent incompatibility groups were IncFIB (K)_Kpn3 (356 genomes, 6.84%), followed by IncI1_Alpha (275 genomes, 5.28%) and IncA/C2 (241 genomes, 4.63%). Plasmids with two replicons (*n* = 1505) showed a higher prevalence of IncFIB(AP001918) (317 genomes, 21.06%), followed by IncHI2A (256 genomes, 17.01%) and IncHI2 (255 genomes, 16.94%). These results demonstrated a higher diversity of incompatibility groups among plasmids with multiple replicons (Table 1).

Among resistant plasmids with a single replicon (*n* = 2,839), the most frequent groups were IncFIB (K)_Kpn3 (195 genomes, 6.86%), followed by IncI1_Alpha (181 genomes, 6.37%) and IncFIB (AP001918) (122 genomes, 4.29%). In contrast, plasmids with two replicons (*n* = 889) showed a higher prevalence of IncFIA (AP001918) (205 genomes, 23.05%) and IncHI2A (149 genomes, 16.76%) (Table 2).

### 3.3 Bacterial Source and Geographic Distribution of Antimicrobial‐Resistant Plasmids

**Figure 1:**
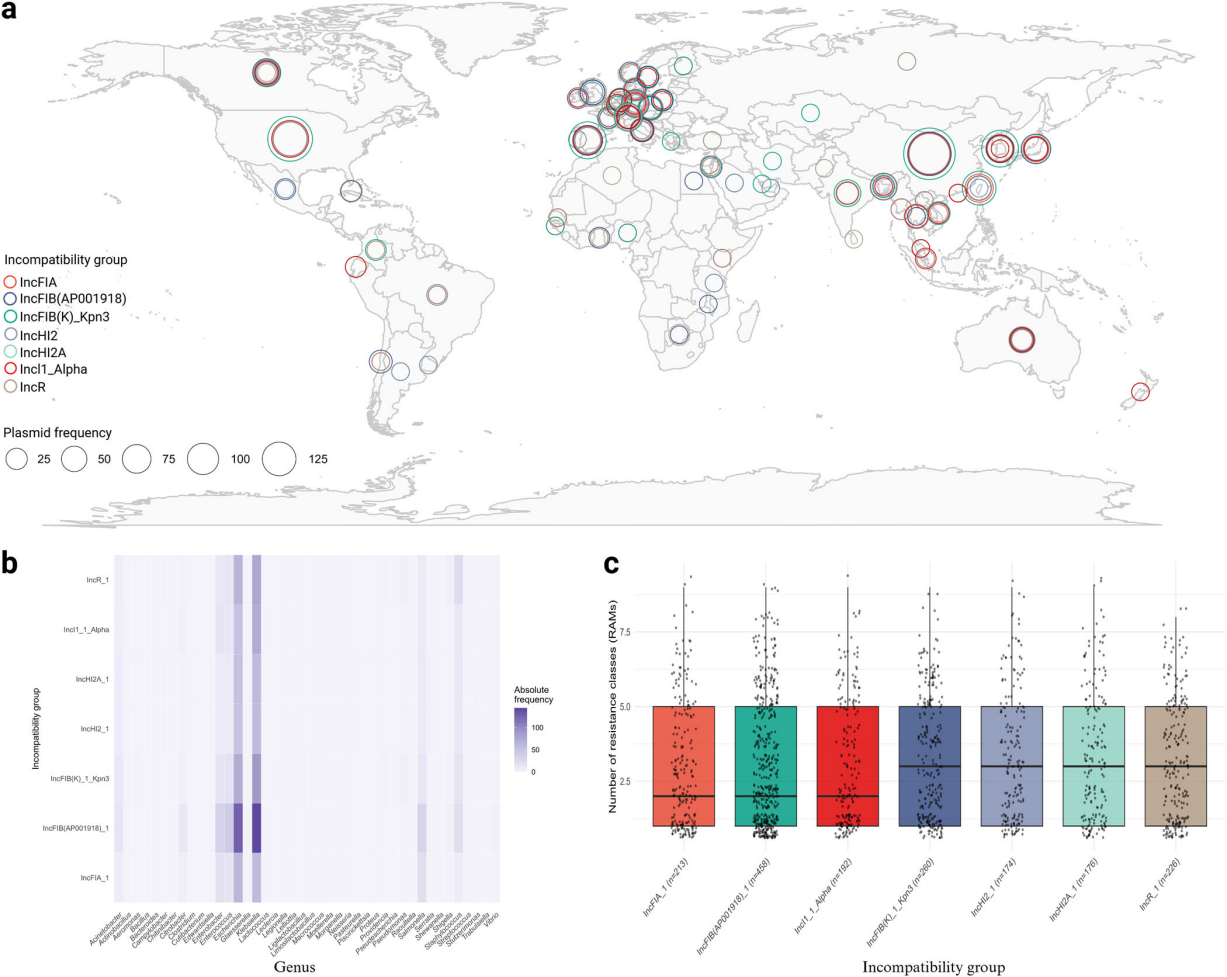
Integrated analysis of the seven most prevalent plasmid incompatibility groups among resistant sequences*.* (a) World map showing the geographic distribution of plasmid frequencies by country, highlighting the groups IncFIB(AP001918), IncFIB(K)_Kpn3, IncR, IncFIA, IncI1_Alpha, IncHI2A, and IncHI2. Circle size is proportional to the number of occurrences; (b) Heatmap of the relative distribution of these groups by bacterial genus, with darker shades indicating higher frequencies; (c) Association between each incompatibility group and the number of antimicrobial classes to which the plasmids confer resistance. Each dot represents an individual plasmid, and the black line indicates the median per group.

We performed a focused analysis on the 4013 resistant plasmids. Data were synthesized based on the most prevalent incompatibility groups, using the criterion of at least 4% of the total, as established in the previous analysis. We selected the most frequent incompatibility groups among the resistant sequences, which were IncFIB (AP01918), identified in 458 sequences (11.4%), followed by IncFIB(K)_Kpn3 in 260 sequences (6.5%), IncR in 226 sequences (5.6%), IncFIA in 213 sequences (5.3%), IncI1_Alpha in 192 sequences (4.78%), IncHI2A in 176 sequences (4.39%), and IncHI2, observed in 174 sequences (4.34%). Initially, the geographic distribution of the plasmid types identified globally was evaluated (Figure 1a). Based on plasmid‐associated metadata, we analyzed the distribution of bacterial genera across the seven main incompatibility groups (Figure 1b). To further quantify host range, we calculated host richness (number of unique genera) and the Shannon diversity index for each incompatibility group. Both metrics were also normalized by the total number of plasmids per group to account for differences in group sizes. Host range varied among incompatibility groups, with IncHI2_1, IncHI2A_1, and IncI1_Alpha exhibiting relatively high diversity, whereas IncFIB(AP01918)_1, despite having the highest number of plasmids, exhibited lower host diversity. Detailed values for host richness and Shannon diversity, both absolute and normalized, are provided in Supporting Information S1: Table S1. Additionally, the quantitative distribution of bacterial genera and species across the incompatibility groups can be observed in Supporting Information S1: Tables S2 and S3, respectively. We also investigated the relationship of these groups with multidrug resistance phenotypes, defined as the simultaneous presence of resistance genes to three or more antimicrobial classes (Figure 1c).

**Figure 2:**
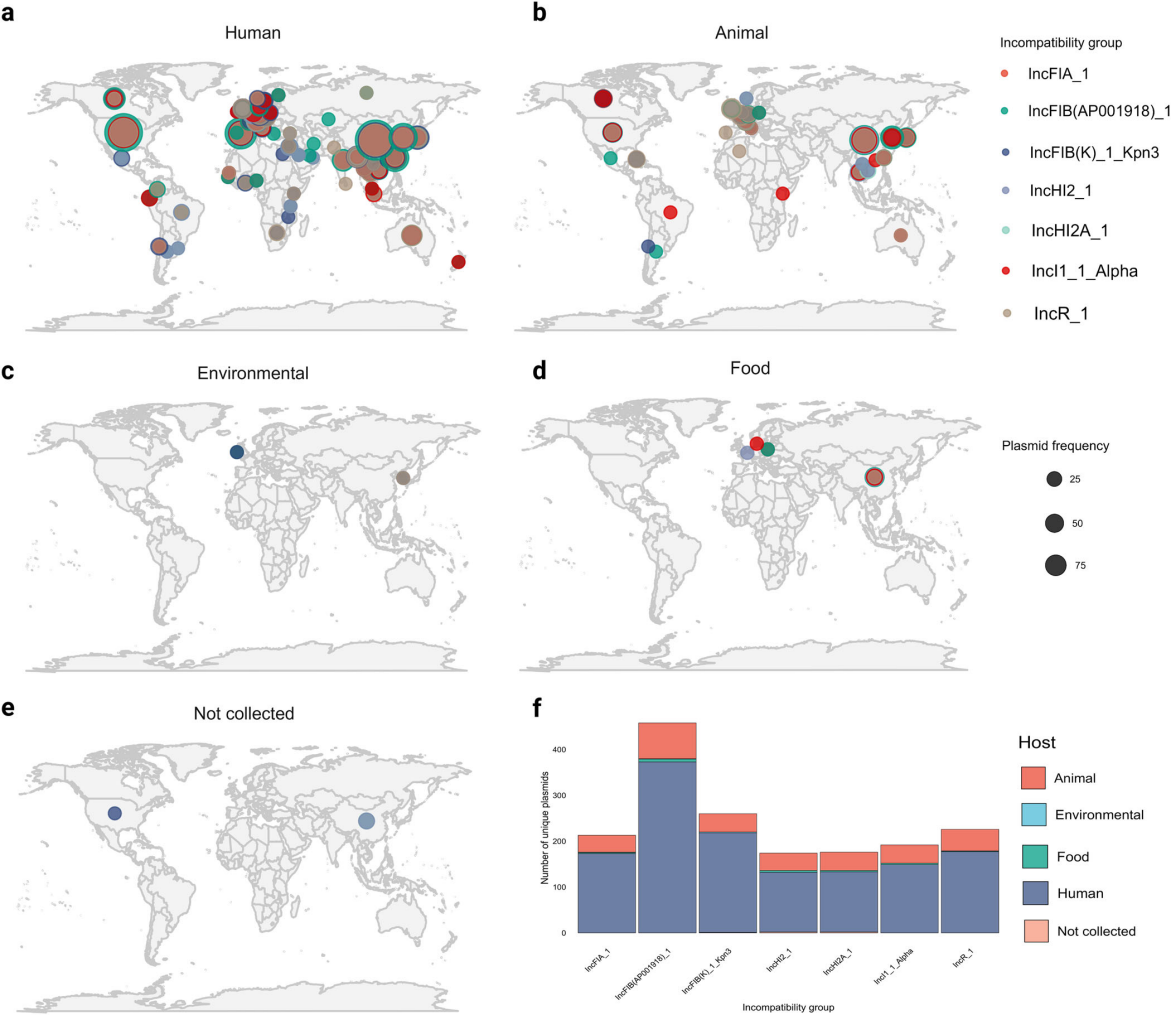
Global distribution of the most prevalent plasmid incompatibility groups according to sample source. Geographic representation of plasmids carrying the seven most frequent incompatibility groups, categorized by isolation source: (a) human, (b) animal, (c) environmental, (d) food, and (e) not reported. Circle size is proportional to the frequency of occurrence per country. (f) Overall distribution of plasmids by incompatibility group, emphasizing the relative contribution of each sample source.

Furthermore, the distribution of incompatibility profiles was evaluated according to the source of bacterial isolation, encompassing samples of human origin (Figure 2a), animal (Figure 2b), environmental (Figure 2c), food (Figure 2d), and those with no available information (Figure 2d). Finally, an overview of the distribution among all sources was displayed (Figure 2f).

### 3.4 Comparative Analysis of AMR Genes and Incompatibility Clusters

#### 3.4.1 The Overview of the Relationship Between AMR and Incompatibility Group

**Figure 3:**
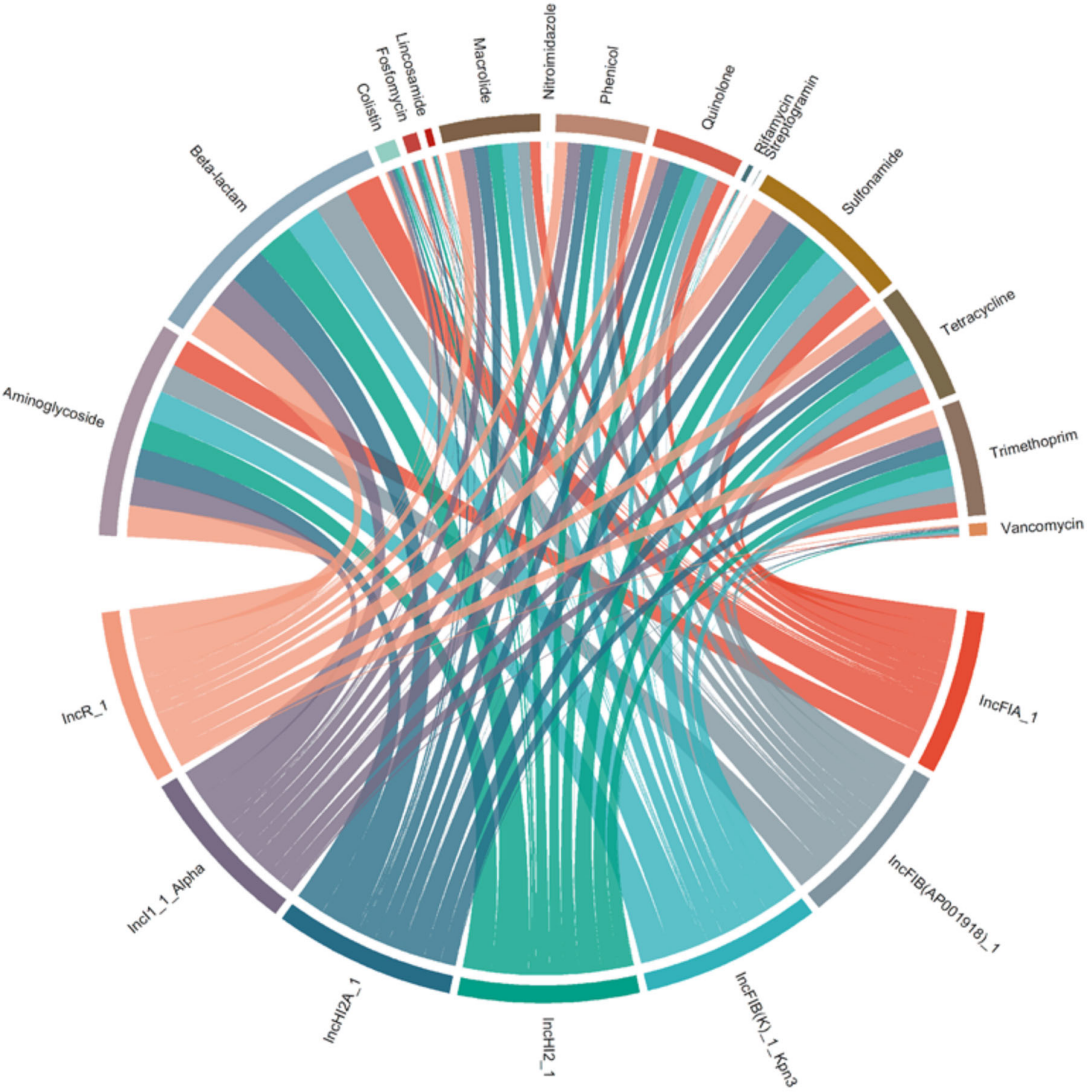
The overview of relationships between antimicrobial resistance and incompatibility group. Each color represents a plasmid incompatibility group, while the connections between the groups and the antimicrobial classes indicate resistance to different antimicrobials. The thickness of the chords in the diagram refers to the frequency of associations between the incompatibility groups and resistance to different classes of antimicrobials.

We investigated the presence of resistance genes in each plasmid, associating them directly with the corresponding incompatibility group, regardless of the number of replicons. The most frequent incompatibility groups among the resistant sequences were IncFIB (AP01918), identified in 458 sequences (11.4%), followed by IncFIB(K)_Kpn3 in 260 sequences (6.5%), IncR in 226 sequences (5.6%), IncFIA in 213 sequences (5.3%), IncI1_Alpha in 192 sequences (4.78%), IncHI2A in 176 sequences (4.39%), and IncHI2, observed in 174 sequences (4.34%). Data were normalized to avoid bias due to variations in the number of sequences between incompatibility groups. The evaluation of resistance distribution was performed by calculating the relative frequency of resistance genes associated with each antibiotic class within each incompatibility group, allowing the comparison of proportions rather than absolute numbers. The calculation was the number of sequences resistant to a given class of antibiotics divided by the total number of sequences within the respective incompatibility group (Figure 3). Furthermore, to investigate possible significant associations between plasmid incompatibility groups (Inc) and antibiotic classes, the proportion of resistant plasmids for each Inc‐class combination was calculated. Statistical association tests were applied: the chi‐squared test for combinations with adequate counts and the simulated Fisher's exact test (10,000 replicates) for combinations with expected values less than five. The results, presented in Supporting Information S1: Table S4, showed that few combinations presented isolated statistical significance. However, no clear and consistent associations were observed between plasmid groups and antibiotic classes.

### 3.5 Relationship Between Antimicrobial Resistance and the Incompatibility Group of Single‐Replicon Resistant Plasmids

**Figure 4:**
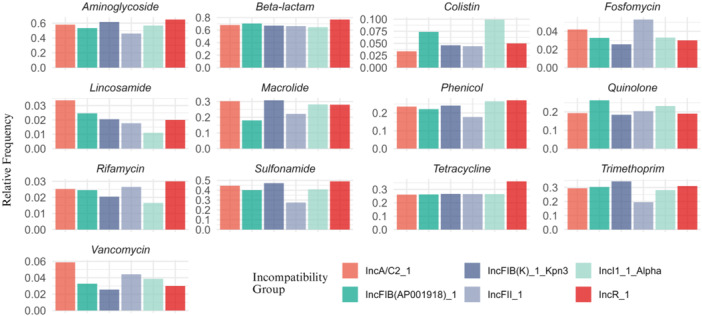
Relative frequency of resistance genes associated with the seven most prevalent incompatibility groups in single‐replicon plasmids, categorized by antimicrobial class. Each bar chart represents one antimicrobial class, and the colors denote different incompatibility groups. Proportions were normalized per group to allow comparative analysis of resistome profiles across plasmids.

Multiple replicon plasmids may have characteristics that distort the observed relationship between incompatibility groups and resistance genes. Hence, we also focused on plasmids with a single replicon to ensure accuracy and reduce potential biases introduced by plasmids with multiple replicons, allowing for a more straightforward and comparable analysis (Figure 4). Among plasmids with a single replicon, the most prevalent incompatibility groups were IncFIB(K)_Kpn3 (195 genomes, 7%), IncI1Alpha (181 genomes, 6%), IncFIB(AP001918) (122 genomes, 4%), IncA/C2 (119 genomes, 4%), IncFII (113 genomes, 4%), and IncR (100 genomes, 4%) (Table 2).

### 3.6 Relationship Between AMR and the Number of Replicons

**Figure 5:**
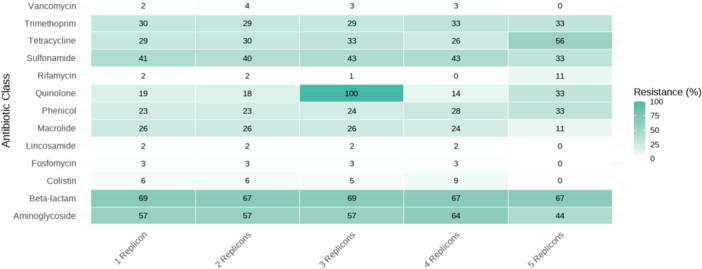
Heatmap showing the relative frequency (%) of resistance to different antimicrobial classes in plasmids carrying 1–5 replicons. Each cell represents the percentage of plasmids harboring resistance genes for the corresponding antimicrobial class, according to the number of replicons. Plasmids with higher replicon counts tend to exhibit broader resistance profiles, suggesting a link between replicon complexity and multidrug resistance.

The analysis revealed a distinct relationship between the number of replicons in plasmids and their AMR genes. Among the 8044 plasmids with a single replicon, 2839 (35.3%) exhibited resistance to at least one antimicrobial. For plasmids with two replicons (2394 sequences), a slightly higher proportion (889 plasmids, 37.1%) carried resistance genes. However, for three replicons (654 plasmids), the proportion decreased to 33.3% (218 plasmids). Interestingly, plasmids with four replicons (*n* = 181) showed a similar trend, with 58 (32.0%) exhibiting resistance. Among the 15 plasmids with five replicons, a notably higher proportion (nine plasmids, 60.0%) contained resistance genes, suggesting that highly complex plasmids might be specialized in acquiring and maintaining resistance. This trend highlights a subtle relationship, where the structural complexity of plasmids (measured by the number of replicons) does not linearly correlate with the likelihood of resistance. To further explore this relationship, we analyzed the associations between replicon categories and resistance to specific antimicrobial classes (Figure 5). In the figure, the heatmap shows the relative frequency of associations between replicon categories and AMR classes, demonstrating the patterns of resistance distribution.

### 3.7 PCA

**Figure 6:**
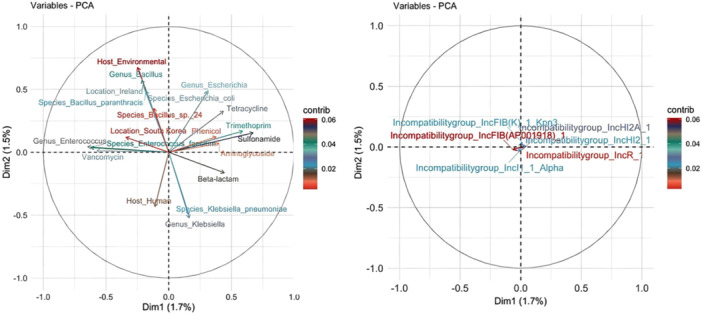
Principal component analysis (PCA) of variables related to resistant sequences. The graph on the left: Graphical representation of the contribution of variables, which include: the host of bacterial isolation (human, animal, and environment), bacterial genus and species, geographic location, and antimicrobial resistance classes. The arrows show the contribution of the variables to the principal components (Dim1 and Dim2). The graph on the right: The contribution of incompatibility groups to the principal components. The color gradient indicates the intensity of the contribution of each variable to the principal components.

PCA was applied to the resistant sequence data set (*n* = 1699) from the major incompatibility groups to identify specific trends and associations within the data, particularly between incompatibility groups and specific AMR classes. The analysis incorporated additional metadata, including isolation source (human, animal, or environmental), geographic origin, and bacterial genus and species. Notably, the contributions of incompatibility groups to the principal components were minimal, with PC1 and PC2 explaining only 1.7% and 1.5% of the total variance, respectively. This indicates that other variables in the data set, such as isolation source, bacterial genus, or geographic location, have a greater influence on the observed variability. In the corresponding PCA plot, the short vectors near the origin further illustrate the limited influence of incompatibility groups on the principal components. These findings align with previous results, reinforcing the weak association between incompatibility groups and specific AMR profiles (Figure 6).

## 4 Discussion

The characterization of plasmids and the identification of their incompatibility groups are fundamental to understanding the patterns of dissemination of resistance mediated by these mobile genetic elements (Rozwandowicz et al. 2018). With advances in *in silico* genome analysis of genomes deposited in large databases, it has become possible to investigate the relationship between plasmids and resistance on a global scale, overcoming the limitations of studies with small sample sizes and significant biases (Wang et al. 2018). Moreover, the PlasmidFinder software is a robust tool for identifying and typing replicons in whole genome sequencing (WGS) data, including those deposited in NCBI (Carattoli et al. 2014). However, despite its effectiveness, among the 28,047 plasmids analyzed, incompatibility groups were identified in only 11,288 sequences. This result can be explained by two main factors: (i) the presence of plasmids lacking replication machinery, which makes their typing impossible using tools such as PlasmidFinder (Coluzzi et al. 2022); and (ii) the incompleteness of the database used, which does not yet include all the genetic markers necessary for typing all sequenced plasmids. These limitations highlight the need for continuous updates to plasmid typing databases and careful interpretation of results, ensuring that subsequent analyses of plasmid diversity and resistance dissemination are even more accurate and comprehensive.

The IncF group was the most frequent among the plasmids typed by PlasmidFinder, present in 55.4% of the genomes analyzed. IncF plasmids, especially the IncFIB (AP001918), IncFIB(K)_Kpn3, IncFIA, and IncFII subgroups, were frequently associated with human isolates, which highlights their clinical relevance (Figure 2f). The result was consistent with previous studies indicating the predominance of the IncF group in isolates from human and animal sources (Rozwandowicz et al. 2018). This plasmid group is also mentioned for its ability to cointegrate with other replicons, such as IncI and IncN, increasing its frequency, especially among mosaic plasmids (Villa et al. 2010; Osborn et al. 2000).

A comparative analysis between plasmids with and without antimicrobial resistance genes showed that the IncFIB replicon (AP001918) was more frequent among resistant plasmids, surpassing IncA/C2, which predominated among non‐resistant plasmids. This difference highlights the strong association between IncF replicons and the spread of AMR genes. Previous studies confirm this association, such as Snaith et al. (2023), who identified 23 IncF plasmids among 40 resistance plasmids isolated from ESBL‐producing *E. coli*. The predominance of IncF plasmids, especially in multi‐resistant isolates (Figure 1c), suggests that these plasmids possess excellent adaptive capacity, allowing their dissemination in various bacterial and human environments (Phan et al. 2015). The diversity of samples analyzed here, encompassing various isolation sources, geographic locations, and bacterial genera, underscores the widespread distribution and adaptability of the IncF plasmid family, particularly the IncFIB subgroup, in disseminating AMR (Rocha‐Gracia et al. 2022; Takayama et al. 2020). Despite differences in contextual factors, such as bacterial hosts and environments, plasmids from this family are consistently associated with resistance genes and are frequently found in multi‐resistant isolates (Rafaï et al. 2015). These findings suggest a robust adaptive capacity of IncF plasmids, enabling their persistence across diverse ecological and clinical settings.

The IncI plasmid group, particularly the IncI1Alpha subgroup, was identified as the second most frequent among resistant and non‐resistant plasmids. These plasmids are well‐documented in isolates from human and farm animal sources, with a notable prevalence in clinically significant pathogens, such as *Escherichia coli* and *Salmonella enterica (*García‐Fernández et al. 2008 *)*. Their role in disseminating resistance genes, especially those conferring resistance to extended‐spectrum cephalosporins, has been widely recognized. This persistence can be attributed to two key factors: their highly efficient horizontal transfer capacity and the minimal metabolic burden they impose on bacterial hosts (Johnson et al. 2015). For instance, the curation of an IncI1 plasmid in *Salmonella enterica* revealed no significant metabolic cost associated with plasmid carriage, indicating its adaptability and maintenance in diverse bacterial populations (Freire Martín et al. 2016). Furthermore, studies have shown that even in the presence of other plasmids, such as IncA/C, the acquisition of IncI1 plasmids does not significantly increase the metabolic burden on bacterial hosts, further facilitating their widespread distribution (Freire Martín et al. 2016).

Interestingly, plasmids classified as “rep” were frequently identified among those with multiple replicons. This classification arises because the “Inc” designation, which refers to plasmid incompatibility groups, is specifically related to the ability of different plasmids to coexist within the same bacterial cell. In contrast, the “rep” classification focuses solely on the replication sequences of plasmids, without addressing their incompatibility characteristics (Johnson and Nolan 2009). In PlasmidFinder, these entries typically correspond to replication initiator sequences from Gram‐positive plasmids. Unlike the “Inc” classification, established for Gram‐negative plasmids and based on well‐characterized incompatibility groups, the “rep” designation reflects replication mechanisms without assigning incompatibility traits. Although a formal incompatibility grouping system is lacking for Gram‐positive plasmids, studies have explored the molecular basis of their exclusion mechanisms, particularly in Staphylococcus species (Kwong et al. 2017).

Several studies have demonstrated a co‐occurrence between plasmid incompatibility groups and the presence of resistance genes associated with certain antimicrobials or antibiotic classes. For instance, Carattoli et al. (2015) identified an association between IncL/M plasmids and ESBL resistance genes, while Foley et al. (2021) highlighted the role of IncA/C and IncI families in disseminating resistance to extended‐spectrum cephalosporins (Carattoli et al. 2015; Foley et al. 2021). Similarly, Rozwandowicz et al. (2018) supported the idea that specific incompatibility groups are linked to particular resistance classes (Rozwandowicz et al. 2018). Based on these findings, we hypothesized that a broad‐scale plasmid genome analysis could provide more robust information regarding the association between specific resistance classes and particular incompatibility groups. Accordingly, our study explored the relationships between the main incompatibility groups found in resistant plasmids (including those with multiple replicons) and resistance to various antimicrobial classes. An additional analysis focused exclusively on plasmids with single replicons to provide a more direct comparison between each incompatibility group and resistance to specific antimicrobial classes. In both analyses, similar results were obtained, with no visually clear associations being observed between the most common incompatibility groups IncFIB(K)_Kpn3, IncI1Alpha, IncFIB(AP001918), IncA/C2, IncFII, and IncR and specific AMR classes. This lack of significant correlations suggests that the relationship between plasmid incompatibility groups and resistance genes may not be systematic or inherent to the incompatibility groups themselves. Instead, these associations may be influenced by external factors such as local selective pressures, bacterial diversity, and geographic variability (Svara and Rankin 2011; Pal et al. 2015). These findings point to the complexity of plasmid‐mediated resistance and emphasize the need for further studies to better understand how contextual factors are linked to the distribution and impact of resistance genes.

PCA was conducted to further explore potential associations within the data set, particularly between plasmid incompatibility groups and AMR. This analysis focused on the most frequent incompatibility groups among resistant plasmids (*n* = 4013) and incorporated additional metadata, including resistance classes, bacterial genus and species, host origin (human, animal, or environmental), and geographic location. The results consistently demonstrated an absence of significant trends linking plasmid incompatibility groups to specific resistance classes or related metadata. These findings suggest that our broader and more comprehensive analyses may have diluted the correlations observed in smaller and context‐specific studies (Carattoli et al. 2015; Foley et al. 2021; Rozwandowicz et al. 2018). For instance, studies with limited geographic scope or narrowly focused bacterial populations often report correlations driven by localized selective pressures, such as antimicrobial usage in specific environments (Kim et al. 2011; Marcadé et al. 2009). Such pressures may result in patterns that are less representative of global trends, emphasizing associations that reflect unique environmental and contextual factors rather than generalizable relationships.

Finally, a higher proportion of resistance genes was observed in plasmids with five replicons compared to those with fewer replicons. This finding aligns with the established role of genetic plasticity, which increases with the number of replicons, enabling the acquisition and retention of diverse AMR genes (Baharoglu et al. 2013). The cointegration of replicons enhances genetic plasticity by allowing plasmids to incorporate various mobile genetic elements, such as transposons and integrons, which facilitate the rapid dissemination of resistance genes across bacterial populations (Partridge et al. 2018). This structural advantage not only promotes the survival of plasmids in diverse environments but also accelerates the spread of resistance genes in the presence of selective pressure. These findings emphasize the evolutionary advantage conferred by multi‐replicon structures, which likely support the adaptability and persistence of these plasmids in challenging environments where resistance genes are advantageous.

Our analysis, enabled by a large‐scale genomic data set, provided a comprehensive understanding of the dynamics between plasmid structure and AMR dissemination. This study emphasizes the role of multi‐replicon plasmids in driving the spread of resistance genes, underscoring their critical contribution to the growing challenge of multidrug‐resistant bacterial populations. Addressing this issue will require targeted interventions aimed at understanding and mitigating the mechanisms by which multi‐replicon plasmids contribute to resistance gene propagation.

## 5 Concluding Remarks

Overall, we found a relation between the number of replicons in plasmids and an increased capacity for integrating AMR genes, demonstrating the critical role of multi‐replicon plasmids in disseminating resistance genes. Although no visually clear association was observed between incompatibility groups and specific antimicrobial resistance classes, the high frequency of IncF, IncH, and IncI replicon families in resistance‐integrating plasmids highlights their importance as key vectors in horizontal gene transfer. This study emphasizes the need to monitor and characterize plasmids with multiple replicons to develop strategies for controlling AMR. Additionally, it identifies potential incompatibility groups as targets for alternative therapeutic interventions to combat AMR.

## Author Contributions

**Hannay Crystynah Almeida de Souza:** conceptualization, data curation, investigation, methodology, writing – original draft, writing – review and editing, visualization. **Pedro Panzenhagen:** conceptualization, investigation, methodology, writing – review and editing. **Anamaria Mota Pereira dos Santos:** conceptualization, investigation, writing – review and editing. **Ana Beatriz Portes:** investigation, writing – review. **Arlen Carvalho de Oliveira Almeida:** statistical analysis, writing – review and editing, and editing, visualization. **Carlos Adam Conte Junior:** funding acquisition, project administration, supervision, writing – review and editing.

## Ethics Statement

The authors have nothing to report.

## Conflicts of Interest

The authors declare no conflicts of interest.

## Supporting information


**Supporting Table S1:** Absolute and normalized values of host richness and Shannon diversity. **Supporting Table S2:** Quantitative distribution of bacterial genera among incompatibility groups. **Supporting Table S3:** Quantitative distribution of bacterial species among incompatibility groups. **Supporting Table S4:** Chi‐square test and Fisher's exact test.
